# Biphasic Calcium Phosphate Biomaterials: Stem Cell-Derived Osteoinduction or In Vivo Osteoconduction? Novel Insights in Maxillary Sinus Augmentation by Advanced Imaging

**DOI:** 10.3390/ma14092159

**Published:** 2021-04-23

**Authors:** Giovanna Iezzi, Antonio Scarano, Luca Valbonetti, Serena Mazzoni, Michele Furlani, Carlo Mangano, Aurelio Muttini, Mario Raspanti, Barbara Barboni, Adriano Piattelli, Alessandra Giuliani

**Affiliations:** 1 Department of Medical, Oral and Biotechnological Sciences, Dental School, University G. D’Annunzio of Chieti-Pescara, 66100 Chieti, CH, Italy; gio.iezzi@unich.it (G.I.); ascarano@unich.it (A.S.); apiattelli@unich.it (A.P.); 2 Faculty of Biosciences and Technology for Food, Agriculture and Environment, University of Teramo, 64100 Teramo, TE, Italy; lvalbonetti@unite.it (L.V.); amuttini@unite.it (A.M.); bbarboni@unite.it (B.B.); 3 Institute of Biochemistry and Cell Biology (CNR-IBBC/EMMA/Infrafrontier/IMPC), National Research Council, Monterotondo Scalo, 00015 Rome, RM, Italy; 4 Department of Clinical Science, Polytechnic University of Marche, Via Brecce Bianche, 60131 Ancona, AN, Italy; sere.mazzoni@gmail.com (S.M.); m.furlani@pm.univpm.it (M.F.); 5 Independent Researcher, 22015 Gravedona, CO, Italy; camangan@gmail.com; 6 Department of Medicine and Surgery, University of Insubria, Via Guicciardini 9, 21100 Varese, VA, Italy; mario.raspanti@uninsubria.it; 7 Fondazione Villaserena per la Ricerca, 65013 Città S. Angelo, PE, Italy; 8 Casa di Cura Villa Serena del Dott. L. Petruzzi, 65013 Città S. Angelo, PE, Italy

**Keywords:** osteoconduction, osteoinduction, biomaterial, biphasic calcium phosphate, rapid prototyping, stem cells, maxillary sinus augmentation, imaging, histology, high-resolution tomography

## Abstract

Maxillary sinus augmentation is often necessary prior to implantology procedure, in particular in cases of atrophic posterior maxilla. In this context, bone substitute biomaterials made of biphasic calcium phosphates, produced by three-dimensional additive manufacturing were shown to be highly biocompatible with an efficient osteoconductivity, especially when combined with cell-based tissue engineering. Thus, in the present research, osteoinduction and osteoconduction properties of biphasic calcium-phosphate constructs made by direct rapid prototyping and engineered with ovine-derived amniotic epithelial cells or amniotic fluid cells were evaluated. More in details, this preclinical study was performed using adult sheep targeted to receive scaffold alone (CTR), oAFSMC, or oAEC engineered constructs. The grafted sinuses were explanted at 90 days and a cross-linked experimental approach based on Synchrotron Radiation microCT and histology analysis was performed on the complete set of samples. The study, performed taking into account the distance from native surrounding bone, demonstrated that no significant differences occurred in bone regeneration between oAEC-, oAFMSC-cultured, and Ctr samples and that there was a predominant action of the osteoconduction versus the stem cells osteo-induction. Indeed, it was proven that the newly formed bone amount and distribution decreased from the side of contact scaffold/native bone toward the bulk of the scaffold itself, with almost constant values of morphometric descriptors in volumes more than 1 mm from the border.

## 1. Introduction

Maxillary sinus augmentation is often necessary prior to implantology procedure, in particular in cases of atrophic posterior maxilla. Different bone regeneration methods were tested over the last 30 years [1,2]. Indeed, despite the fact that autologous bone is still considered the gold standard grafting material, because of the presence of viable osteoblasts, organic and inorganic matrices, and growth factors, its limited availability and the requirement of additional surgical procedure, with a possible increase in morbidity [2], has promoted several research studies focused on testing alternative bone substitute biomaterials (BSB). In particular, the risk of disease transmission by allo/xenografts, oriented the research towards synthetic bone substitutes, attempting to reproduce the physical/chemical properties of native bone tissues and achieving osteointegration, osteoconduction, and possibly, osteoinduction [2]. Biphasic calcium phosphates, produced with a balanced combination of hydroxyapatite (HA) and tricalciumphosphate (TCP), which means a stable HA phase and a resorbable TCP [3], are highly biocompatible with an efficient osteoconductivity [4]. Moreover, in the last 10 years, the three-dimensional (3D) additive manufacturing allowed to produce biphasic calcium phosphate scaffolds with a controlled 3D architecture [5,6,7], which was shown to increase their biocompatibility versus commercial products [8,9]. However, almost all synthetic BSBs were shown to require long times to regenerate large portions of bone tissue [9,10]; thus, their use in surgical clinics is still limited and needs further investigation.

Cell-based tissue engineering is considered a promising strategy to support bone healing and regeneration [11,12,13,14]. In this context, placental sites, in particular the amnion, appeared to be particularly interesting because they represent reserves of stem/progenitor cells, especially promising for therapeutic applications [15,16,17,18]. More recently, amniotic membranes have been investigated as a possible source of stem/progenitor cells for therapeutic applications. In particular, amniotic epithelial cells (AEC) and amniotic fluid mesenchymal stem cells (AFMSC) can be obtained without any ethical limitation [16,19]. Moreover, recently, AECs and AFMSC were both used in preclinical settings of sinus lift [20,21] to evaluate if they could represent an alternative source of progenitor/stem cells for cell therapies in dentistry. In these studies, ovine-derived amniotic derived cells of different origin (from the epithelial layer or fluid) were seeded on a custom-made biphasic calcium phosphate scaffold or on a commercial magnesium-enriched hydroxyapatite (MgHA)/collagen-based material, respectively, and grafted into the maxillary sinus of adult sheep. Cell influence on bone regeneration was evaluated 45 and 90 days after grafting. In particular, ovine AECs (oAECs) were shown to have a modulatory role in angiogenesis, vascular endothelial growth factor expression, and inflammation management, suggesting also that oAECs were able to directly participate in the process of bone deposition [20].

However, controversial results have been reported in literature when augmented sites, with or without cell support, were compared with naturally healed sites [22,23,24]. This fact may be due to the use of two-dimensional (2D) diagnostic techniques, like histology and electron microscopy. Thus, the high variability of these data reported in literature suggested coupling these techniques with advanced three-dimensional (3D) quantitative methods [25].

In the framework of the 3D imaging techniques, X-ray microtomography (microCT) was shown to be one of the most powerful tools for the BSB characterization, both in vitro [26,27,28] and after grafting in vivo [29,30]. By microCT it is not only possible to achieve bone microarchitecture and local mineralization analysis [29,30], but also to assess bone ultrastructure [31], also providing quantitative information on the construct structure. Moreover, the use of synchrotron radiation-based microCT has introduced numerous advantages in the analysis versus conventional laboratory-based microCT devices because of the synchrotron production of X-rays with higher beam intensity, higher spatial coherence, and monochromaticity. Beam hardening effects are significantly reduced, making much more accurate the segmentation process of the images and, in turn, the reliability of the whole quantitative analysis [26,31].

In the present research, osteoinduction and osteoconduction properties of biphasic (30%HA–70%TCP) calcium-phosphate construct made by direct rapid prototyping (rPT) [20] engineered with ovine-derived amniotic epithelial cells (oAEC) or amniotic fluid cells (oAFMSC) were evaluated 90 days after grafting in a validated sinus augmentation experimental model. To this aim, a cross-linked experimental approach based on Synchrotron Radiation microCT and histology analyses was performed.

## 2. Materials and Methods

### 2.1. Preclinical Study Ethical Issues

The present study was approved by the Ethics Committee of the Universities of Teramo and Chieti-Pescara (prot. 05/2012/CEISA/PROG/32) since the animal preclinical experimets were carried out before the national entry into force of D. lgs. 26/2014 on the protection of animals used for scientific purposes adopting the EU directive 63/2010.

More in details, the preclinical study was performed using 10 adult sheep, 2 years old, and 40–50 kg of weight targeted to receive scaffold alone (CTR), oAFSMC, or oAEC engineered constructs. The sheep were bred according to the European community guidelines (E.D. 2010/63/UE). After 2 weeks of animal quarantine, surgical procedures were carried out in an authorized veterinary hospital. The animals were followed daily in the postsurgical period and the evolution of sinus transplantation was documented weekly. The animals were euthanized to explant grafted sinuses at 90 days.

### 2.2. Study Overview

The preclinical setting were performed according to Barboni et al. [20]. Briefly, ovine-derived stem cells from amniotic membrane (oAEC) and from amniotic fluid (oAFMSC) were collected by three amniotic membranes derived from slaughterhoused pregnant sheep at middle stage of gestation. The timing of gestation was deduced by fetus length [32].

These cells were seeded at the final concentration of 2 × 10^6^ onto a 3D scaffold composed by hydroxyapatite (HA) and β-tricalcium phosphate (β-TCP) in a ratio of 30/70.

The trasplantation settings were performed by grafting:

1. Ctr-group: Nr = 4 HA + TCP scaffolds (no stem cell loading);
2. oAEC-group: Nr = 3 HA + TCP scaffolds seeded with amniotic epithelial-derived cells;
3. oAFMSC-group: Nr = 3 HA + TCP scaffolds seeded with meschymal stem cells extracted from amniotic fluid.

The samples were analyzed by histology and synchrotron radiation-based microtomography (SR-microCT) after the permanence in vivo in the sheep for 3 months.

### 2.3. Scaffold Fabrication

The ceramic scaffolds used in this study were fabricated by the direct rapid prototyping technique dispense-plotting (Biomed Center, Bayreuth, Germany) [6,7,33]. A virtual scaffold model was designed with a cylindrical outer geometry by using 3D computer aided design software. The inner geometry, i.e., the pathway of the material rods, was defined by custom-made software, which generates the control commands of the rapid prototyping machine. To build up the green bodies, material rods consisting of a paste-like aqueous ceramic slurry were extruded out of a cartridge through a nozzle and deposited using an industrial robot (GLT, Pforzheim, Germany). In this study, hydroxyapatite (HA) and tricalcium phosphate (TCP) powders (Merck, Darmstadt, Germany) were blended to get a biphasic powder mixture with a HA/TCP weight ratio of 30/70. The rod deposition was controlled in x, y, and z direction to assemble 3D scaffolds layer by layer on a building platform. The assemblies made of ceramic slurry were dried at room temperature and subsequently sintered at 1250 °C for 1 h.

Afterwards, the sintered scaffolds were cut in small blocks with a volume of about 0.14 cm^3^.

### 2.4. Ovine AEC and AFMSC Characterization and Stem Cell Scaffold Loading

In order to compare the osteo-regeneration potential of amniotic-derived cells under allotransplantation settings, 3D scaffolds were loaded with 2 × 10^6^ oAEC or oAFMSC. The amniotic-derived cells were isolated and characterized according to Refs [20,21].

More in detail, freshly isolated oAEC were isolated, amplified, and tested for cytokeratin 8 and adhesion molecules (CD166, CD29) positivity and for alpha smooth muscle actin (mesodermal marker), hemopoietic and the two major histocompatibility complexes (MHCI and II) negativity accordingly to previously validated protocols [20].

Ovine AFMSC were derived from each fetus and screened by flow cytometry in order to evaluate hemopoietic markers (CD14, CD31, and CD45), adhesion molecules (CD49f, CD29, and CD166), stemness intracellular markers (OCT4, SOX2, Nanog, and TERT), and MHC class I and II antigen expressions as previously described [21].

Both typologies of cells were seeded on single blocks of synthetic bone substitute (∼0.14 cm^3^) and incubated in a 35 mm Petri dish over a roller apparatus (Wheaton, Millville, NJ, USA) using a validated protocol [21]. Each culture was taken under agitation for 3 days at a speed of 6 rpm. The scaffolds used in control group (CTR) were incubated without cells for 3 days under analogous cultural conditions.

### 2.5. Sinus Augmentation Experimental Animal Model

Sheep were operated under general anesthesia to carry out a bilateral sinus augmentation as previously described in Berardinelli et al. [21]. Prior to flap local anesthesia was obtained with Articaine^®^ (Pierrel Pharma, Italy) associated with epinephrine 1:100,000. A triangular flap was elevated with a flap elevator that had to be adherent to the bone so that the periosteum remained undamaged. The first incision was made on the top of the alveolar ridge horizontally with a relieving incision in the mesial and distal regions of sinus. A second incision was made in the distal region without a mesial relieving incision. Full-thickness flaps were elevated to expose the alveolar crest and the lateral wall of the maxillary sinus. The lateral wall of the sinus was approached through an oval ostectomy (1 cm up and 1 cm caudal to tuber facial tuberosity) carried out using a piezoelectric unit (Vario-Surgery NSK, Tochigi, Japan) using a tip under cold (4–5 °C) sterile saline irrigation. The Schneiderian membrane was elevated with curettes of different shapes, until it became completely detached from the lateral, inferior, and medial walls of the sinus. The space under sinus membrane was filled with two blocks of biomaterial alone (CTR) or previously loaded with PHK26 labeled oAEC. All flaps were carefully sutured with resorbable suture (Glicofil Lac^®^, Assut, Magliano dei Marsi, Italy). No membranes perforation or laceration was recorded. The animals were treated i.v. with 20 mg/kg of ampicillin (Vetamplius^®^, Fatro, Italy) every 12 h for 3 days. Surgical wounds were inspected daily. The animals were euthanized at 90 days p.i. by applying an overdose of thiopental (Pentothal Sodium, Intervet, Segrate, Italy) and embutramide (Tanax^®^, Intervet, Segrate, Italy).

### 2.6. Histological Analysis

The biopsies were fixed by immediate immersion in 10% buffered formalin and processed (Precise 1 Automated System; Assing, Rome, Italy) to obtain thin ground sections, as previously described [34]. The specimens were dehydrated in an ascending series of alcohol rinses and embedded in glycol-methacrylate resin (Technovit 7200 VLC; Kulzer, Wehrheim, Germany).

After polymerization, the specimens were sectioned, along their transversal axis, with a high precision diamond disk at about 150 µm and ground down to about 30 µm with a specially designed grinding machine Precise 1 Automated System [34]. Three slices were obtained from each specimen, subsequently stained with acid fuchsin and toluidine blue before the analysis. Histological analysis was carried out using a light microscope (Laborlux S, Leitz, Wetzlar, Germany) connected to a high-resolution video camera (3CCD, JVCKY-F55B, JVC, Yokohama, Japan) and interfaced with a monitor and PC (Intel Pentium III 1200 MMX, Intel, Santa Clara, CA, USA). This optical system was associated with a digitizing pad (Matrix Vision GmbH, Oppenweiler, Germany) and a histomorphometry software package with image capturing capabilities (Image-Pro Plus 4.5, Media Cybernetics Inc., Immagini & Computer Snc, Milano, Italy).

### 2.7. Synchrotron Radiation-Based Micro-CT Analysis

The X-ray tomographic experiments were performed at the SYRMEP beamline (ELETTRA synchrotron light source, Trieste, Italy). The radiographs were acquired at an X-ray energy of 23 keV; sample-detector distance of 5 cm; pixel size of 4.1 µm; 1800 projections per sample over 180°; exposition time per projection: 2.5 s.

The reconstruction of the tomographic slices was carried out using a custom-developed software [35], applying the standard filtered back-projection algorithm.

The commercial software VG Studio MAX 1.2 (Volume Graphics, Heidelberg, Germany) was used to generate 3D images and visualize the phase distribution in 3D. Optimal image quality settings were obtained using the Scatter HQ algorithm with oversampling factor of 5.0. X-ray contrast differences within samples translated into different peaks in the grey level scale, corresponding to the different phases. Three peaks could clearly be distinguished (background, newly formed bone—in the samples investigated after implantation, and scaffold material). The mixture modeling algorithm (NIH ImageJ Plugin) was implemented to threshold these histograms; therefore, the representative threshold values were set to 85 (newly formed bone) and 155 (scaffold).

A structural analysis of the trabecular structure (including scaffold and newly formed bone phases) was performed, mapping the whole samples in order to verify if oAECs or oAFMSCs had induced morphometric modification on the structures. The following morphometric parameters were evaluated in the overall mineralized structure (scaffold + newly formed bone): Structure Volume (SV) to Total Volume (TV) ratio (SV/TV-expressed as a percentage), Structure Surface to Structure Volume ratio (SS/SV-per millimeter), Structure Thickness (STh-expressed in micrometers), Structure Number (SNr-per millimeter), Anisotropy Degree (DA), and Connectivity Density, i.e., number of trabeculae per unit volume (Conn.D.-expressed in µm^−3^). The Degree of Anisotropy (DA) is a measure on how highly oriented the substructures are within a certain volume; indeed, trabecular structures could vary their orientation depending on stem cell culture and could become anisotropic. The mean intercept length (MIL) method for determining ani-sotropy was used. DA was equal to *1-(length of the shortest axis/length of the longest axis),* resulting in 0 = fully isotropic structure; 1 = fully anisotropic structure.

Particular care was also devoted to the quantitative analysis of the regenerated bone using the same quantitative descriptors applied to the overall mineralized structure: Bone Volume to Total Volume ratio (BV/TV-expressed as a percentage) and regenerated Bone Thickness (BTh-expressed in micrometers).

Moreover, analogue quantitative descriptors were also applied to the residuals of the scaffold after 3 months of grafting in vivo: Scaffold Volume to Total Volume ratio (ScV/TV-expressed as a percentage) and Scaffold Thickness (ScTh-expressed in micrometers).

### 2.8. Statistical Analysis

The statistical analysis of the morphometric microCT data was performed using the software package SigmaStat 3.5 (Systat, San Jose, CA, USA). All values were expressed as mean and standard deviations. The sample groups were compared with analysis of variance (one-way ANOVA); all pairwise multiple comparisons were performed by the Holm–Sidak method, considering a *p*-value < 0.05 to be statistically significant.

## 3. Results

### 3.1. Histological Analysis

**Figure 1:**
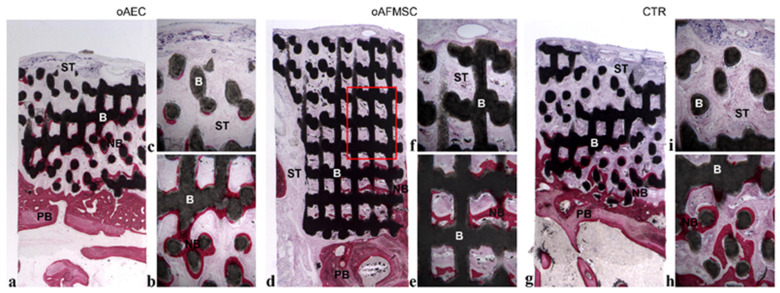
Histological analysis. (**a**,**d**,**g**) At low magnification, the samples of all groups showed the residual biomaterial blocks (B) in contact with the pre-existing bone (PB) at the bottom of the samples, where new bone formation (NB) was present. At the top of the samples, soft tissue (ST) was detected; (**d**) a square, highlighted in red, showed the porous and intact structure of the biomaterial block (Acid fuchsin-Toluidine blue 9X). (**b**,**e**,**h**) At the bottom of the sample, the biomaterial blocks (B) were in close contact with the preexisting bone (PB); in these portions, the new bone formation (NB) was observed (Acid fuchsin-Toluidine blue 40X). (**c**,**f**,**i**) At the top of the sample, soft tissue (ST) within the biomaterial block was observed (Acid fuchsin-Toluidine blue 40X).

At low magnification, the samples of all groups showed the residual biomaterial block in contact with the pre-existing bone, that partially retained its interconnected porous microstructures (Figure 1a,d,g).

Specifically, only at the bottom of the sample, where the biomaterial blocks were in close contact with the preexisting bone, new bone formation was observed in some areas. The biomaterial showed signs of resorption, and, in fact, the microporous structure was not clearly detected (Figure 1b,e,h). Regarding the behavior of the biomaterial, it underwent resorption in some fields, appearing as residual particles surrounded by the newly formed bone. Indeed, in some areas, osteoblasts depositing osteoid matrix were detected.

In the portions far from the pre-existing bone, soft tissue within the biomaterial block was observed (Figure 1c,f,i).

Regarding the bone formation, there were no substantial differences between the groups. However, no signs of inflammation were present in the test groups compared to the control group.

### 3.2. MicroCT Analysis

We investigated a single biopsy for each sheep because, in order to not introduce sources of variability, the scaffolds were grafted exactly in the same region for each sheep. A sampling 3D reconstruction is made available for a oAEC-cultured scaffold as Supplementary Material (Video S1). Morphometric analysis obtained mapping the whole samples, as described in Table 1, apparently showed better performances of the oAEC-cultured scaffolds than oAFMSC- and Ctr-cultured ones. In particular, a higher BTh of struts was found in oAEC-cultured scaffolds than in oAFMSC- and Ctr-cultured scaffolds. Moreover, the overall STh and ScTh were shown to be significantly higher in oAEC-cultured scaffolds than in oAFMSC- and in Ctr-cultured scaffolds. Furthermore, Conn.D. was proved to be reduced in oAEC-cultured scaffolds versus oAFMSC- and Ctr-cultured scaffolds.

**Figure 2:**
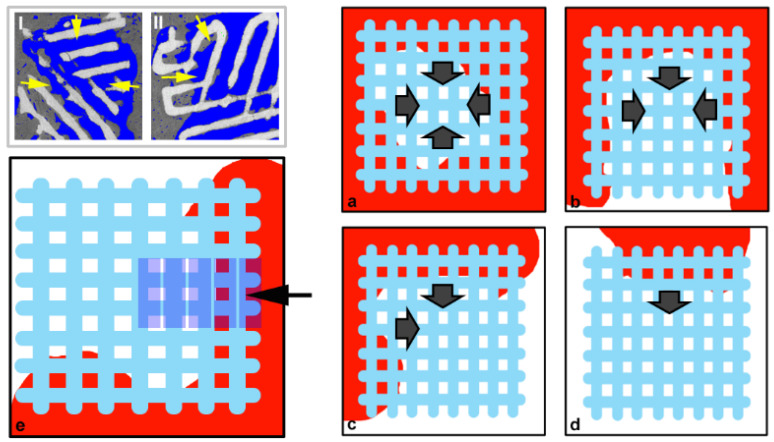
Study approach of the morphometric parameters vs. distance from native surrounding bone. (I–II) Sampling slices of casual samples: yellow arrows indicate strict contact of the scaffold with surrounding native bone. Dark grey: bone; light grey: scaffold. (**a**–**d**) Possible sample settings: (**a**) Native bone fully surrounds the scaffold; (**b**) Native bone surrounds the scaffold from three sides; (**c**) Native bone surrounds the scaffold from two sides; (**d**) Native bone surrounds the scaffold from only one side. (**e**) Concept for the selection of the Volumes of Interest (VoIs): 5 VoIs of 1.6 × 1.6 (base) × 0.4 (thickness) mm^3^ were selected, the first centered in one side where the scaffold was in strict contact with the native bone, the following going towards the bulk of the scaffold. Red: bone; light blue: scaffold.

The analysis was continued, checking if the previous morphometric results were re-liable or affected by the specific scaffold dimension/distance to the native surrounding bone tissue. In other words, it was verified if predominant was the action of the seeded stem cells or the osteoconduction action of native surrounding bone. This result was achieved approaching the study of the morphometric parameters vs. distance from native surrounding bone, as shown in Figure 2.

**Figure 3:**
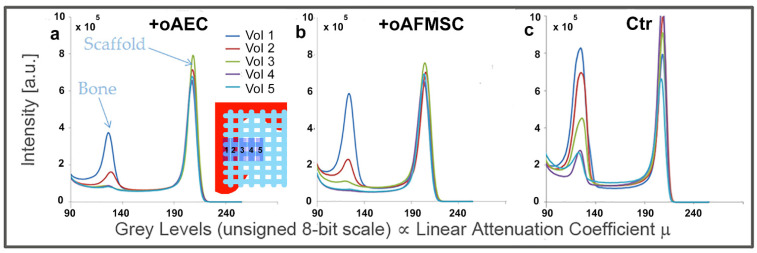
Profiles of the intensity counts vs. gray levels (proportional to the linear attenuation coefficient µ) in representative samples of each group. The integrated areas of the represented peaks correspond to the newly formed bone (left peak) and to the scaffold (right peak) (**a**) in the oAEC-group, (**b**) in the oAFMSC-group, and (**c**) in the Ctr-group.

Briefly, starting from the scaffold side in strict contact with the native bone and going towards the bulk of the scaffold, five volumes of interest (VoIs), each with a volume of 1.6 × 1.6 (base) × 0.4 (thickness) mm^3^, were selected and studied. It was demonstrated that bone regeneration depended on the distance from the surrounding native bone, as shown in the histograms of Figure 3.

**Figure 4:**
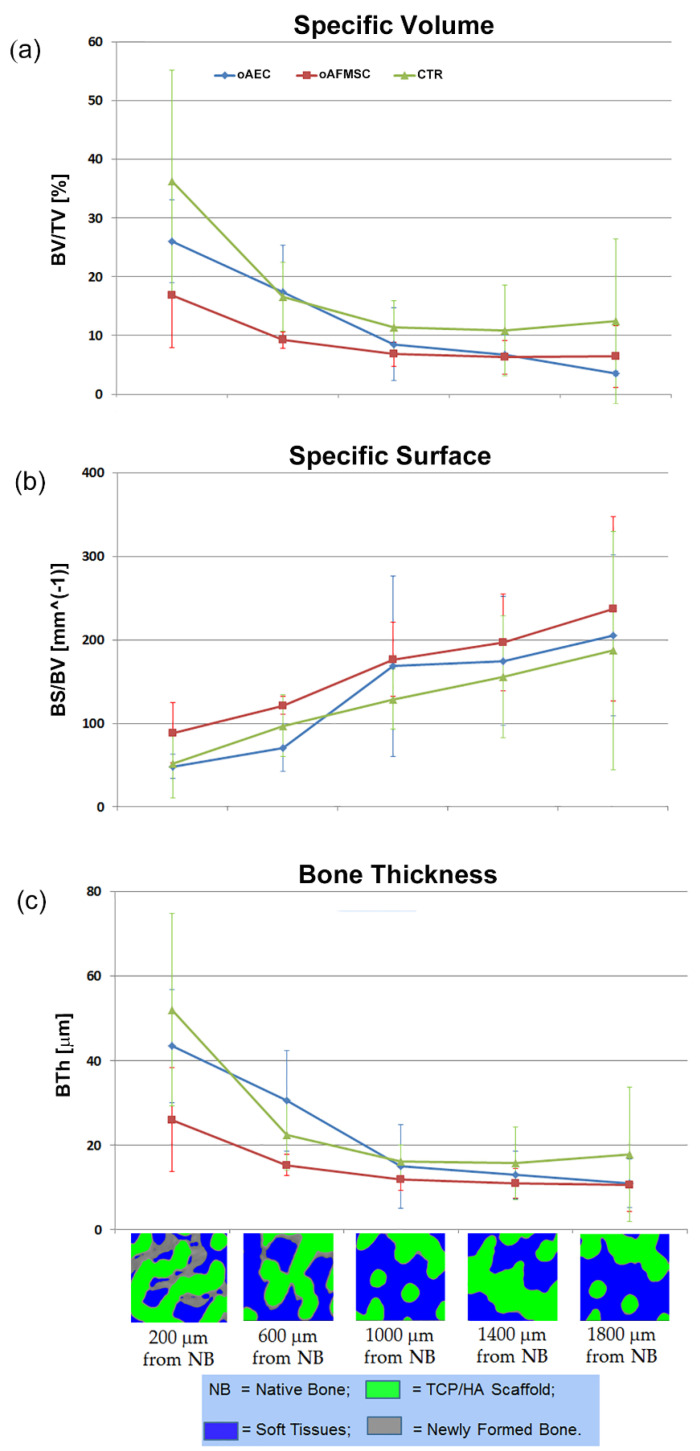
Morphometric analysis of the newly formed bone in the 5 VoIs, from the scaffold surface towards the bulk: (**a**) Specific Volume (BV/TV); (**b**) Specific Surface (BS/BV); and (**c**) Bone Thickness (BTh). For each group of study, mean value between samples and standard deviations are reported for each VoI. Bottom images: sampling microCT 2D-slices at the different depths. Grey: newly formed bone; Green: scaffold; Blue: soft tissues.

Indeed, it was shown for one representative sample for each group that the further the volume is from the contact surface with the native bone, the less is the amount of regenerated bone. Therefore, the osteoconductive properties of these constructs were studied; in particular, each VoI was morphologically investigated using some of descriptors previously introduced, i.e., the specific bone volume (BV/TV—Figure 4a), the specific bone surface (BS/BV—Figure 4b), and the newly formed bone thickness (BTh—Figure 4c).

The predominant action of the surrounding native bone osteoconduction versus the stem cell-inducted osteogenesis was confirmed by the fact that no significant differences were detected between oAEC-, oAFMSC-seeded, and Ctr scaffolds in terms of BV/TV, BTh, and BS/BV trends. Moreover, it was shown that the osteoconduction was effective up to more than 1.0 mm from the surrounding native bone. Indeed, BV/TV and BTh decreased and, coherently, BS/BV increased up to the depth of around 1 mm; there, the profiles reached a plateau and remained constant towards the bulk of the scaffold.

## 4. Discussion

The present research aimed to study and discriminate osteoinduction and osteoconduction properties in constructs made of biphasic (30%HA–70%TCP) calcium phosphate scaffolds, produced by direct rapid prototyping (rPT) and loaded with ovine-derived amniotic epithelial cells (oAEC) or amniotic fluid cells (oAFMSC). The experimental approach was based on the combined use of Synchrotron Radiation microCT and histology. The study was focused on samples retrieved from an ovine animal model after the permanence for 90 days in maxillary defects; indeed, our focus was the analysis of newly formed mineralized extracellular matrix, while previous studies [20,21] already clarified that, at 45 days from grafting, oAEC and oAFMSC-loaded scaffolds already showed reduced fibrotic reaction, a limited inflammatory response, and an accelerated process of angiogenesis.

Inside the error due to the limited sample size, the present study further confirmed, with new analytical methods, the high biocompatibility of biphasic (30%HA–70%TCP) calcium-phosphate scaffolds; it demonstrated the high degree of integration of this scaffold with surrounding bone tissues and the osteogenic influence provided in grafted sinuses.

On the other hands, the present data supported the idea that both sources of amniotic derived cells were eligible for tissue engineering strategies applied to maxillofacial surgery, on the basis of their potential to support bone integration by promoting vascularization [20], as a consequence of their long-term survival when allotransplanted.

The present morphometric study, addressed to describe the average descriptors obtained by mapping the entire volumes of the investigated samples, seemed to show that the regenerated bone was significantly thicker in oAEC-cultured scaffolds than in oAFMSC- and in Ctr-cultured scaffolds. However, the study performed taking into account the distance from native surrounding bone demonstrated something quite different, i.e., that no significant differences occur in bone regeneration between oAEC-, oAFMSC-cultured, and Ctr samples and that there is a predominant action of the osteoconduction versus the stem cells osteoinduction. Indeed, it was proved that the newly formed bone amount and distribution decreased from the side of contact scaffold/native bone toward the bulk of the scaffold itself, with almost constant values of morphometric descriptors in volumes more than 1 mm from the border.

This may be due to multiple factors. Several authors agree that bone substitutes, in which thickness exceeds 400 μm, need to be vascularized in vitro before grafting them in vivo to achieve cellular survival [36]. Moreover, it was demonstrated both in vitro and in vivo that the healing process is normally delayed in the presence of biomaterials seeded with stem cells with respect to the cases of the same biomaterial but seeded with differentiated cells, most likely because stem cells growing onto the scaffold have not only to adhere and proliferate like differentiated cells but also to activate the differentiation process and all these processes need more time [27,28,36,37,38,39].

Thus, this study once again highlighted the fundamental role of biphasic calcium phosphates (BCPs), in particular those produced by rapid prototyping technology, in promoting osteoconduction. In terms of translation of rapid prototyping technology of BCP-scaffolds, it has to be stressed that the reconstruction of complex bone defects requires biomaterials that enable the fabrication of customized implants for patient. In this direction, rapid prototyping techniques are excellent methods to produce scaffolds with a complex internal or external structure based on tomographic data. Several in vivo studies, including the present one, documented the ability of BCP structures to promote bone ingrowth and remodeling as well as vascularization [20,21]. The success of these biphasic products as bone substitutes was the combination of the higher solubility of ß-TCP with the lower solubility of HA, involving promotion of early bone ingrowth [40]. 3D printed samples are characterized by a high microporosity, which results from the voids between the powder particles, as well as by a macroporosity in the range of 100–300 μm. The properties of the scaffold (shape, mechanical stability, biological behavior) can be optimally adjusted to copy bone tissue, which has to be replaced. For a tissue engineered scaffold, it is important to obtain specific pore size and interconnectivity in 3D. These scaffolds can be engineered to have specific mechanical and material properties that closely approximate those of the tissue to be replaced, providing a delivery vehicle for osteoinductive molecules and/or osteogenic cells and consequently facilitating the bone healing.

Moreover, the present results offer an additional evidence on how sinus augmentation outcome was improved by combining the emerging material techniques with the use of adequate sources of progenitor cells. Since bone regeneration seemed to proceed centripetal after the transplantation of a good synthetic bone substitute, the use of an adequate source of stem cells such as the amniotic derived ones, may impact positively by enhancing the foci of bone nucleation, thus increasing, strengthening, and accelerating the alveolar bone reconstruction [20,21]. Indeed, although the role of amniotic derived cells has been studied in the present work exclusively evaluating on bone deposition, such an effect should also be interpreted as the consequence of a complex process involving different systems all converging in defining the success of tissue regeneration. Most of them are greatly under the control of stem cells such as the modulation of the inflammatory phase [41], the remodeling of extracellular matrix, and the guidance of neuro-and angiogenesis events [15].

This study presented two small limitations, i.e., the limited sample size that possibly could prevent further morphometric differences from being detected, and the resolution of the microCT experiment (4.1 µm) that prevented to detect bone deposits with thicknesses under 5–7 µm when combined, like in this study, with the presence of phase contrast residuals in the microCT images.

## 5. Conclusions

Although the microCT study, obtained on mean morphometric values by mapping the entire volume of the samples examined, seemed to show that the regenerated bone was thicker on structures obtained from oAEC cultures than on those with oAFMSC or Ctr samples, the study carried out taking into account the distance of the analyzed volumes of interest from the native surrounding bone demonstrated that there are no significant differences in terms of bone regeneration between the samples cultured with oAECs, oAFMSCs, or Ctr. This fact demonstrated that, after three months from grafting in sheep, in these constructs, the action of osteoconduction was predominant when compared to the efficiency of stem cells in terms of osteoinduction.

## Figures and Tables

**Table 1:** Three-dimensional morphometric analysis of the mineralized microarchitecture obtained mapping the whole samples. Mean ± standard deviation. Pairwise multiple comparisons considering a *p*-value < 0.05 statistically significant.

Morphometric Parameter	oAEC	oAFMSC	Ctr	Significant Difference (*p* Value)
Total Specific Volume SV/TV—[%]	53.4 ± 5.0	51.0 ± 9.5	49.9 ± 5.5	No: *p* > 0.05
Total Specific Surface SS/SV—[mm^−1^]	23 ± 3	31 ± 10	32 ± 6	No: *p* > 0.05
Total Thickness STh—[µm]	88 ± 11	70 ± 19	64 ± 11	Yes: oAEC vs. Ctr (*p* = 0.014)
Total Struts Nr SNr—[mm^−1^]	6.4 ± 1.3	8.2 ± 3.7	8.5 ± 1.9	No: *p* > 0.05
Anisotropy Degree DA	0.232 ± 0.059	0.198 ± 0.047	0.251 ± 0.077	No: *p* > 0.05
Connectivity Density Conn.D. (×10^−4^)—[µm^−3^]	1.195 ± 0.695	2.015 ± 1.157	3.490 ± 1.967	Yes: oAEC vs. Ctr (*p* = 0.021)
Bone Specific Volume BV/TV—[%]	17.4 ± 5.3	12.5 ± 6.1	13.0 ± 5.0	No: *p* > 0.05
Bone Thickness BTh—[µm]	18 ± 4	9 ± 2	10 ± 3	Yes: oAEC vs. Ctr (*p* < 0.001) oAEC vs. oAFMSC (*p* < 0.001)
Scaffold Specific Volume ScV/TV—[%]	36.1 ± 2.0	38.5 ± 7.0	37.6 ± 2.3	No: *p* > 0.05
Scaffold Thickness ScTh—[µm]	89 ± 7	70 ± 19	68 ± 12	Yes: oAEC vs. Ctr (*p* = 0.007) oAEC vs. oAFMSC (*p* = 0.019)

## Data Availability

Some data are contained within the article or supplementary material. The microCT data presented in this study are available on request from the corresponding author. The microCT data are not publicly available due to their extremely large size.

## Supplementary Materials

The following are available online at https://www.mdpi.com/article/10.3390/ma14092159/s1, Video S1: 3D exploration into a +oAEC-cultured biphasic calcium phosphate scaffold.
